# Increasing New Delhi metallo-β-lactamase-positive *Escherichia coli* among carbapenem non-susceptible *Enterobacteriaceae* in Taiwan during 2016 to 2018

**DOI:** 10.1038/s41598-021-82166-8

**Published:** 2021-01-28

**Authors:** Yu-Shan Huang, Wan-Chen Tsai, Jia-Jie Li, Pao-Yu Chen, Jann-Tay Wang, Ying-Tsong Chen, Feng-Jui Chen, Tsai-Ling Lauderdale, Shan-Chwen Chang

**Affiliations:** 1 Department of Internal Medicine, National Taiwan University Hospital, 7 Chung-Shan South Road, Taipei, 100 Taiwan; 2 Graduate Institute of Clinical Medicine, National Taiwan University College of Medicine, Taipei, Taiwan; 3 Department of Internal Medicine, National Taiwan University Hospital Biomedical Park Hospital, Taipei, Taiwan; 4 Taipei Municipal Jianguo Senior High School, Taipei, Taiwan; 5 Institute of Molecular and Genomic Medicine, National Health Research Institutes, Miaoli, Taiwan; 6 National Institute of Infectious Diseases and Vaccinology, National Health Research Institutes, Miaoli, Taiwan

**Keywords:** Infectious diseases, Bacteria, Clinical microbiology, Microbial genetics

## Abstract

New Delhi metallo-β-lactamase (NDM) had been reported to be the predominant carbapenemase among *Escherichia coli* in Taiwan. However, studies focusing on the clonal background and epidemiology of plasmids carrying NDM genes were limited. Between 2016 and 2018, all clinical *E. coli* and *Klebsiella pneumoniae* isolates that were non-susceptible to ertapenem, meropenem, and imipenem were tested for carbapenemase-encoding genes (CEGs) and antimicrobial susceptibilities. Molecular typing was performed on all carbapenemase-producing isolates. Whole genome sequencing (WGS) was performed on all NDM-positive *E. coli* isolates. Twenty-three (29.5%) of 78 carbapenem non-susceptible *E. coli* and 108 (35.3%) of 306 carbapenem non-susceptible *K. pneumoniae* isolates carried CEGs. The most prevalent CEGs in carbapenemase-producing *E. coli* (CPEc) were *bla*_NDM_ (39.1%) and *bla*_IMP-8_ (30.4%), while that in carbapenemase-producing *K. pneumoniae* was *Klebsiella pneumoniae* carbapenemase (KPC) (72.2%). Fifteen sequence types were identified among 23 CPEc, and 55.6% of NDM-positive *E. coli* isolates belonged to ST410. WGS showed ST410 isolates were highly clonal and similar to those from other countries. All NDM-5-positive *E. coli* isolates carried identical IncX3 plasmid harboring *bla*_NDM-5_ but no other antimicrobial resistance (AMR) genes. In each of the four NDM-1-positive *E. coli* isolates, the *bla*_NDM-1_ was present in a ∼ 300 kb IncHI2/IncHI2A plasmid which carried an array of AMR genes. NDMs are the most prevalent carbapenemase among CPEc in Taiwan. Awareness should be raised as the prevalence of NDM-positive *E. coli* might increase rapidly with IncX3 plasmid and globally distributed strain ST410 being the potential vectors for wide dissemination.

## Introduction

Antimicrobial resistance is a major threat to public health worldwide. Carbapenems have been a last resort treatment option against multidrug-resistant Gram-negative pathogens, including cephalosporinase and/or extended-spectrum β-lactamase-producing *Enterobacteriaceae*^1^. With the wide spread use of carbapenems, the emergence of carbapenem-hydrolyzing β-lactamases has become a challenge to clinicians. Isolates of carbapenemase-producing *Enterobacteriaceae* (CPE) are often multidrug-resistant, leaving limited therapeutic options for treating severe infections.

In line with the increased global burden of CPE, the prevalence of CPE in Taiwan has also increased in recent years^2^, and the production of various carbapenemases are increasingly reported in *Klebsiella pneumoniae* and *Escherichia coli*^3,4^. *E. coli* spreads more readily in community than *K. pneumoniae* and represents the leading cause of both community and nosocomial infections. Therefore, the emergence of carbapenemase-producing *E. coli* (CPEc) raises a special concern of CPE dissemination in community.

New Delhi metallo-β-lactamase (NDM) belongs to the amber class B β-lactamases and is capable of hydrolizing almost all β-lactams except for monobactam^5^. The gene encoding NDM (*bla*_NDM_) is often carried by different types of transferable plasmids, thus facilitate NDM dissemination not only by clonal expansion but also by inter-strain and inter-species horizontal transfer of *bla*_NDM_^6^. In addition, NDM-positive *Enterobacteriaceae* in poultry also contributed to the global spread^7^. In Taiwan, *bla*_NDM_ was first identified from *K. pneumoniae* in 2010 and the first case of NDM-positive *E. coli* infection was reported in 2012^8,9^. Following the first report, a study showed that the incidence of NDM-positive *E. coli* rose sharply and NDM became the predominant carbapenemase among *E. coli* species^10^. However, there have been limited reports focusing on the clonal background of NDM-positive strain and the epidemiology of plasmids carrying *bla*_NDM_, which contribute to the rapid dissemination of NDM.

Before novel and effective antimicrobial agents become available, containment of CPE has become a major public health issue. The present study aimed to strengthen our understanding on the epidemiology of the two most important CPE (*E. coli* and *K. pneumoniae*) in Taiwan and focused on the prevalence of NDM-positive strains.

## Results

### Prevalence of carbapenemase in CnSEc and CnSKP

During the study period, 78 (0.5%) of the 17,018 *E. coli* and 306 (3.1%) of the 9802 *K. pneumoniae* clinical isolates collected at NTUH were carbapenem-non-susceptible. The majority of carbapenem-non-susceptible *E. coli* (CnSEc) was from urine (n = 25, 32.1%) and anal swab specimens (n = 19, 24.4%), while the majority of carbapenem-non-susceptible *K. pneumoniae* (CnSKP) was from urine (n = 86, 28.1%) and respiratory tract specimens (n = 86, 28.1%).

**Figure 1:**
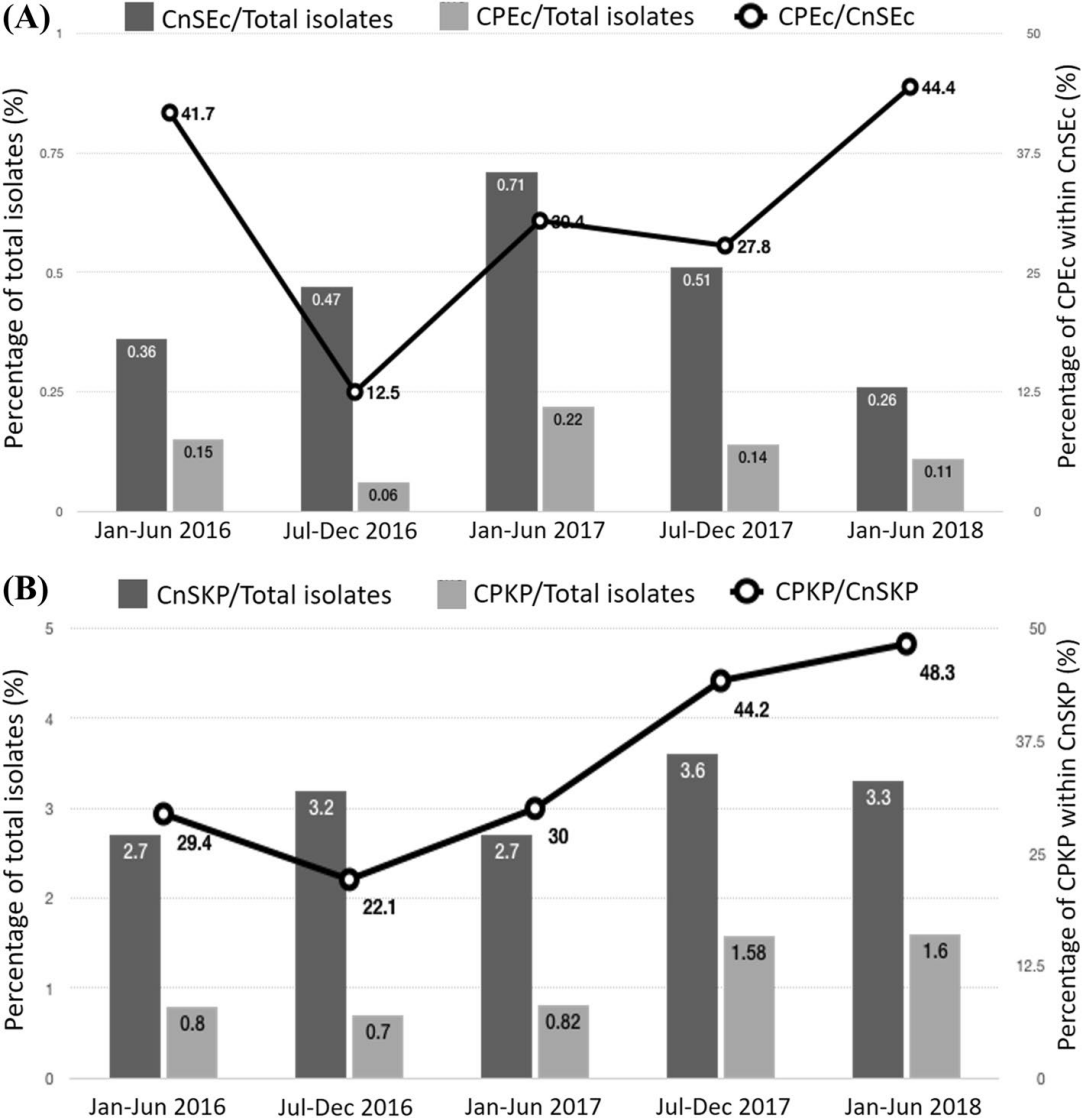
Trend in proportions of (**A**) carbapenem-non-susceptible and carbapenemase-producing *E. coli* (CnSEc and CPEc), and (**B**) carbapenem-non-susceptible and carbapenemase-producing *K. pneumoniae* (CnSKP and CPKP) from January 2016 to June 2018.

Among the 78 CnSEc isolates, 23 (29.5%) were CPEc, including nine (39.1%) NDM, seven (30.4%) IMP-8, five (21.7%) KPC-2, and two (8.7%) VIM-1. Among the 306 CnSKP isolates, 108 (35.3%) were carbapenemase-producing *K. pneumoniae* (CPKP), including two dual carbapenemase-positive *K. pneumoniae*. Among the 106 CPKP isolates with a single carbapenemase, 77 (72.6%) were positive for KPC, 23 (21.7%) were positive for VIM-1, four (3.8%) were positive for IMP-8, and two (1.9%) were positive for NDM (Table 1). The NDM-5-positive CPKP and one NDM-5-positive CPEc were isolated from the same patient one month apart. During the study period, the percentage of CPEc among CnSEc did not increase significantly (*p* = 0.66), while the prevalence of CPKP and the proportion of CPKP among CnSKP increased significantly during the study period (*p* = 0.001) (Fig. 1A,B).

**Table 1:** The distribution of carbapenemases and sequence types of carbapenemase-producing *E. coli* and *K. pneumoniae,* 2016–2018.

Type of carbapenemase	Carbapenemase-producing *E. coli* (n = 23)		Carbapenemase-producing *K. pneumoniae* (n = 108)	
	Carbapenemase, n (%)	Sequence type	Carbapenemase*, n (%)	Sequence type
KPC	5 (21.7)		78 (72.2)	
KPC-2	5	131, 156, 457, 710, 1177	74	11(n = 56), 15 (n = 17), 897
KPC-3	0	–	1	11
KPC-17	0	–	3	11 (n = 2), 690
NDM	9 (39.1)		3 (2.8)	
NDM-1	4	410 (n = 3), 155	0	–
NDM-4	0	–	2	15 (n = 2)
NDM-5	5	38, 167, 359, 410 (n = 2)	1	2889
IMP-8	7 (30.4)	38 (n = 2), 156, 453, 1193, 1670, 3997	5 (4.6)	37, 76, 225, 919, 1537
VIM-1	2 (8.7)	48, 5229	24 (22.2)	14, 20, 34, 196, 225 (n = 9), 322 (n = 2), 736 (n = 2), 1715, 1947 (n = 2), 2163, no match (n = 3)
OXA48	0 (0)	–	0 (0)	–

*Two carbapenemase-producing *K. pneumoniae* isolates were positive for dual carbapenemase: one positive for IMP-8 and VIM-1; the other positive for KPC-2 and NDM-4.

### Antimicrobial susceptibility testing of carbapenem-non-susceptible isolates

The susceptibility of antimicrobial agents and the distribution of MICs are presented in Table 2 and Supplementary Table 1. The carbapenem-non-susceptible isolates were highly resistant to extended spectrum β-lactams. For the non-β-lactams, the susceptibility rate of CnSEc was the highest among tigecycline (97.4%), followed by colistin (94.9%) and amikacin (92.3%). Non-CP-CnSEc isolates were more susceptible to ciprofloxacin and levofloxacin than CPEc. CPEc isolates were 100% susceptible to tigecycline and colistin (Table 2). NDM-positive CPEc were 100% non-susceptible to all β-lactams, and had lower susceptibility to aminoglycosides than IMP-, KPC-, or VIM-positive CPEc (Supplementary Table 2).

**Table 2:** Susceptibility patterns of carbapenem-non-susceptible *E. coli* and *K. pneumoniae* isolates to different antimicrobial agents.

Antimicrobial agents	Carbapenem-non-susceptible *E. coli* (%Susceptible)			Carbapenem-non-susceptible *K. pneumoniae* (%Susceptible)		
	All (n = 78)	Non-CP-CnSEc (n = 55)	CPEc (n = 23)	All (n = 306)	Non-CP-CnSKP (n = 198)	CPKP (n = 108)
Cefotaxime	1.3	1.8	0	0.3	0.5	0
Ceftazidime	1.3	1.8	0	0.3	0.5	0
Cefmetazole	5.1	3.64	8.7	2.6	2.5	2.8
Cefepime	7.7	7.3	8.7	5.6	7.1	2.8
Amikacin	92.3	90.9	95.7	86.6	84.3	90.7
Gentamicin	57.7	60.0	52.2	35.0	34.9	35.2
Ciprofloxacin	32.1	38.2	17.4	14.7	15.7	13.0
Levofloxacin	34.6	40.0	21.7	15.4	16.7	13.0
Tigecycline	97.4	96.4	100	67.9	70.2	63.6
Pip/Tazo	3.9	3.7	4.4	0	0	0
TMP/SMX	33.3	38.2	21.7	22.5	16.2*	34.3*
Colistin	94.9	92.7	100	84.0	86.4	79.6

*CPKP vs. Non-CP-CnSKP, *p* < 0.01.

CPEc, carbapenemase-producing *E. coli*; CPKP, carbapenemase-producing *K. pneumoniae*; MICs, minimal inhibitory concentrations; Non-CP-CnSEc, non-carbapenemase-producing carbapenem-non-susceptible *E. coli;* Non-CP-CnSKP, non-carbapenemase-producing carbapenem-non-susceptible *K. pneumoniae;* Pip/Tazo, piperacillin/tazobactam; TMP/SMX, trimethoprim/sulfamethoxazole.

The susceptibility rate of CnSKP was the highest among amikacin (86.6%), followed by colistin (84.0%) and tigecycline (67.9%). The susceptibility rate between CPKP and non-CP-CnSKP isolates were also analyzed and compared. The most significant difference between these two groups were the higher rates of susceptibility to trimethoprim/sulfamethoxazole among CPKP compared to non-CP-CnSKP (34.3% and 16.2%, *p* < 0.01) (Table 2).

### Clonal relationship of CPEc and CPKP

**Figure 2:**
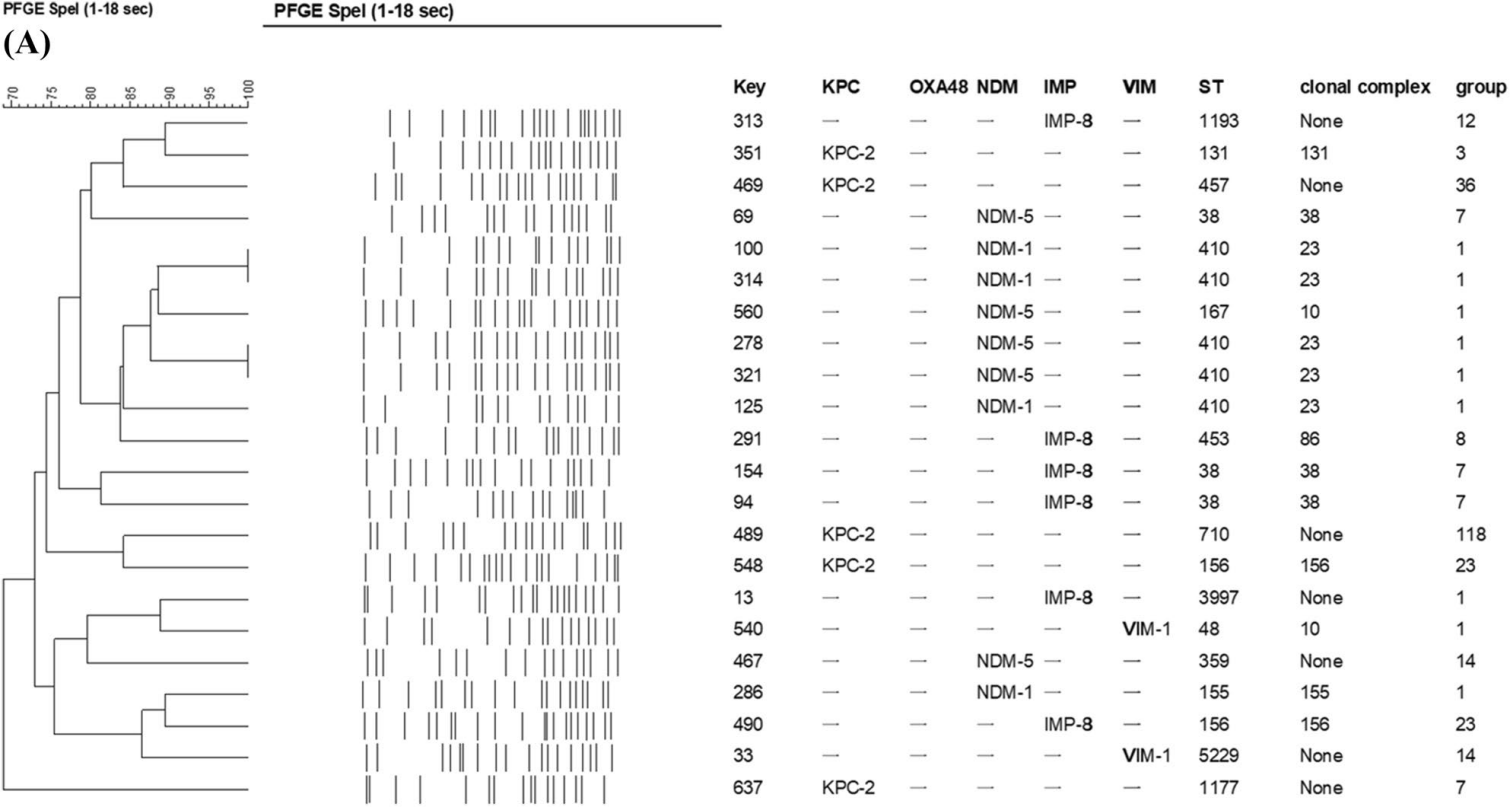

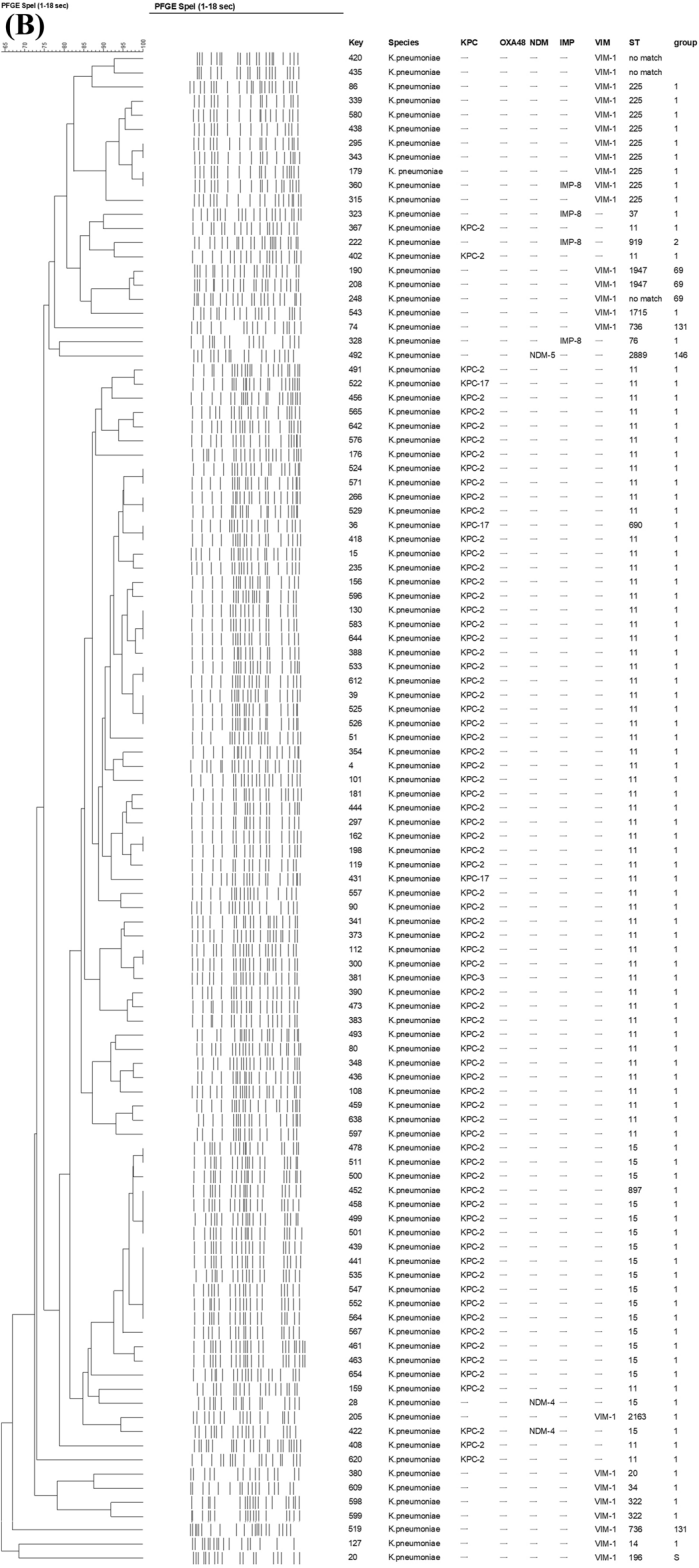
Comparisons of carbapenemase-producing (**A**) *E. coli* and (**B**) *K. pneumoniae* isolates by PFGE dendrogram and MLST.

**Figure 3:**
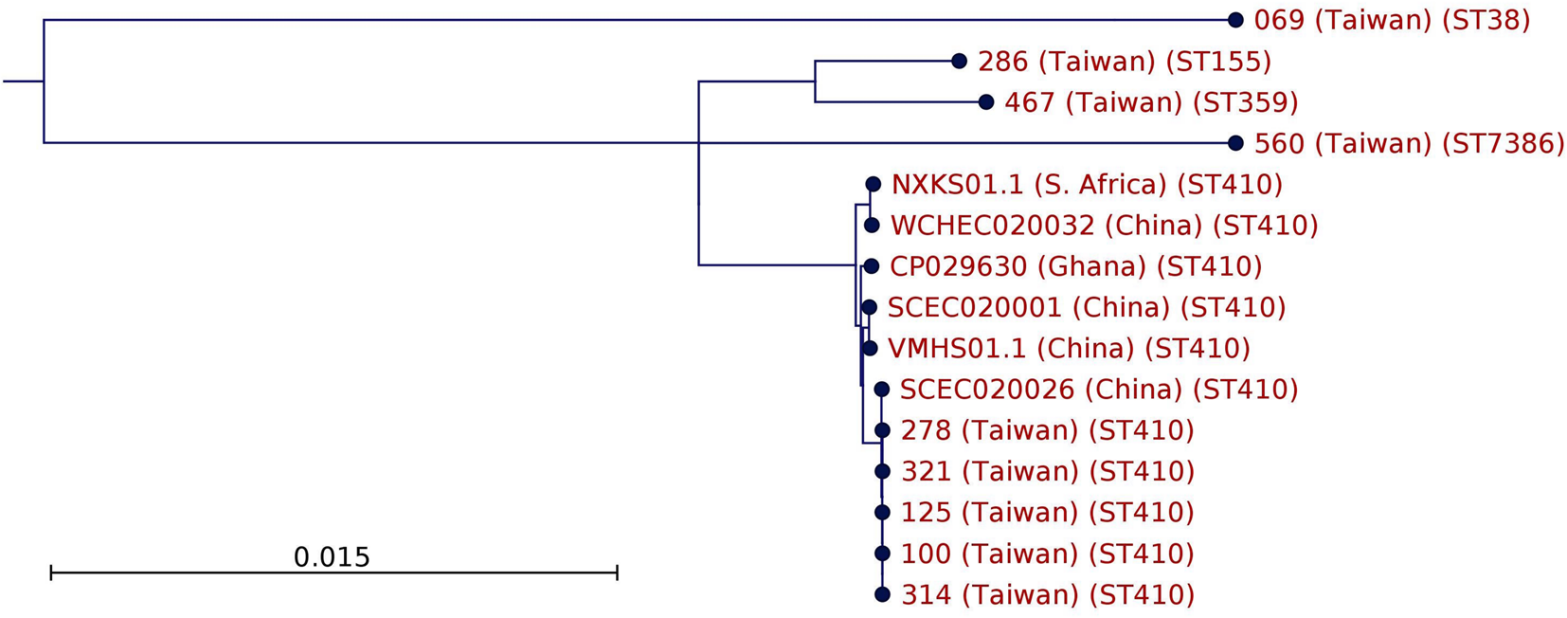
Core genome MLST analysis result based on the whole genome sequencing data on the chromosome of ST410.

The clonal relatedness determined by PFGE and MLST of carbapenem-non-susceptible isolates are shown in Fig. 2A,B. The sequence type of CPEc and CPKP isolates are listed in Table 1. CPEc isolates were highly diverse in terms of sequence type (ST), with a total of 15 STs identified. However, ST410 was the most predominant one among NDM-positive *E. coli* isolates (5/9, 55.6%) and all of them shared > 80% similarity in PFGE banding patterns to each other (Fig. 2A). The remaining NDM-positive *E. coli* belonged to ST38, ST155, ST167, and ST359. The clonal relationship of the CPEc was further evaluated using the WGS data. Core genome MLST (cgMLST) analysis based on the WGS data on the chromosome also showed that our ST410 isolates are nearly identical to each other and highly similar to the ST410 isolates from China, South Africa and Ghana (Fig. 3).

Among CPKP, more than 19 different STs were identified. Most of the KPC-positive *K. pneumoniae* were ST11 (59/78, 75.6%), followed by ST15 (17/78, 21.8%). All CPKP belonging to ST11 were positive for KPC. ST11 accounted for 75.7% (56/74) KPC-2, 100% (1/1) KPC-3 and 66.7% (2/3) KPC-17. The KPC-positive ST11 *K. pneumoniae* could be grouped into 4 main pulsotypes. The KPC-positive ST15 isolates belonging to another distinct pulsotype. The VIM-1 positive *K. pneumoniae* isolates were also distinct from the KPC-positive isolates by PFGE. (Fig. 2B).

### Location and surrounding structure of the NDM-positive *E. coli*

**Figure 4:**
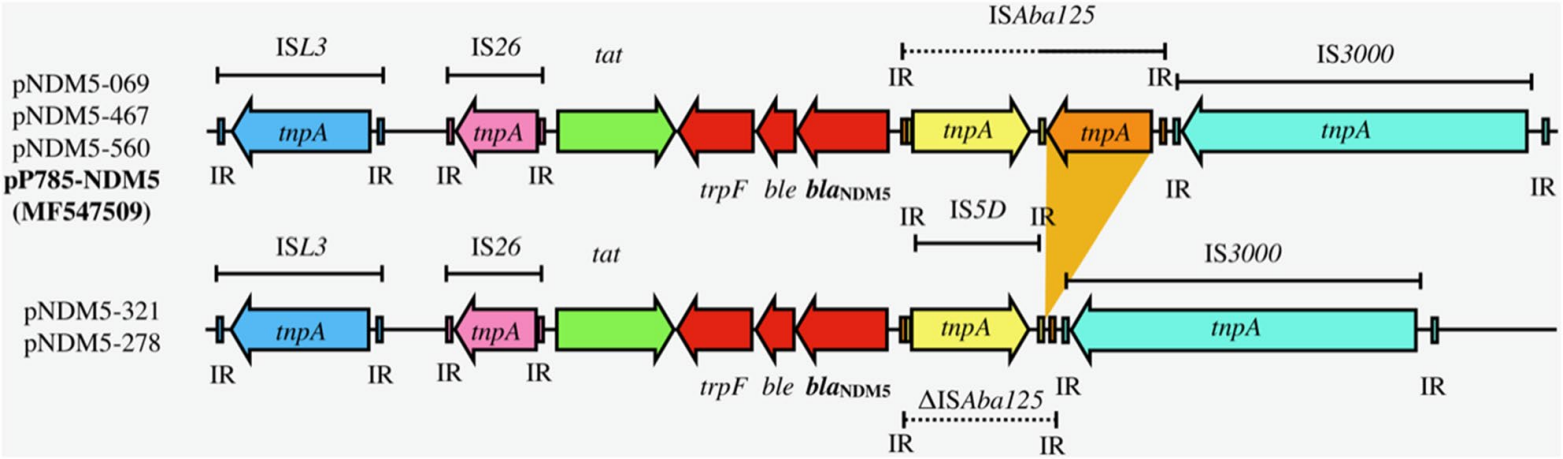
Analysis of IncX3 plasmid sequences harboring bla_NDM-5_ in this study.

In all five NDM-5-positive isolates (069, 278, 321, 467, 568), the NDM-5 gene was located in a ~ 46 kb IncX3 plasmid that are nearly identical to each other. These plasmids are similar to pP785-NDM5 from China (GenBank accession No. MG547511)^11^. The inverted repeat (IR) sequence of the ISAba125 was found at the immediate upstream of the NDM-5 gene (Fig. 4). Same as pP785-NDM5, these plasmids did not carry other antimicrobial resistance (AMR) genes.

**Figure 5:**
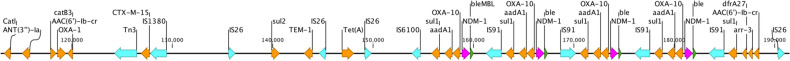
NDM repeat without inverted repeat (IR) in plasmid of NDM-1-positive eco286 isolate.

In each of the 4 NDM-1-positive *E. coli* isolates, the *bla*_NDM-1_ was present in a ~ 300 kb IncHI2/IncHI2A plasmid. These plasmids shared a backbone similar to that of pEc21617-310, a 309-kb plasmid identified in *E. coli* isolate 21617 from Taiwan (unpublished, GenBank accession No. MG878867) (Supplementary Fig. 1). In contrast to the NDM-5-carrying plasmids that harbored no other AMR genes, the NDM-1 plasmids carried an array of AMR genes associated with resistance to multiple classes of antimicrobials, including aminoglycosides [aph(3″)-Ib, aph(6)-Id, aadA1], β-lactams (*bla*_CTX-M-15_, *bla*_OXA-1_, *bla*_TEM-1_), fluoroquinolones [aac(6′)-Ib-cr, qnrB1], chloramphenicol (catA1, catB3), trimethoprim (dfr), sulfonamides (sul), and tetracycline [tet(A)]. Of interest is that in one of the plasmids (isolates 286), the *bla*_NDM-1_ gene was present in more than one copy along with an adjacent bleomycin resistance gene (bleMBL) and IS91 (Fig. 5).

### Carbapenem consumption

Annual carbapenem consumption showed increasing trends during the analysis period, from 34.7 DDD per 1000 patient-day in 2010 to 73.2 DDD per 1000 patient-days in 2018 (*p* = 0.006, Supplementary Fig. 2). There was significant correlation between carbapenem consumption and the rate of CPKP among CnSKP (r = 1.00, *p* < 0.01) while the rate of CPEc among CnSEc (r = 0.60, *p* = 0.28) was insignificant.

## Discussions

Among the carbapenem non-susceptible *Enterobacteriaceae* collected during a three-year period in Taiwan, 29.5% of CnSEc and 35.3% of CnSKP isolates carried carbapenemase-producing genes. The most prevalent carbapenemase in CPEc was NDM (39.1%), while that in CPKP was KPC (72.2%). The sequence type of CPEc was diverse, but five of nine NDM-positive *E. coli* isolates belonged to ST410, and they were highly clonal. In addition, all NDM-5-positive *E.coli* isolates carried identical IncX3 plasmid which harbored *bla*_NDM-5_.

Non-CPE isolates had accounted for the majority of carbapenem-non-susceptible *Enterobacteriaceae* (CnSE) in Taiwan^2^. Combination of Amp-C β-lactamse and loss of outer membrane porin used to be the main mechanism of carbapenem non-susceptibility among *Enterobacteriaceae* isolates in Taiwan^9,10,12^. However, an up-surging CPE trend had been observed, with the prevalence of carbapenemase production increased from 6% in 2010 to 46.5% in 2017 among CnSKP isolates in Taiwan^13–16^.

Carbapenemase-producing isolates were less common in *E. coli* than in *K. pneumoniae*. Among *E. coli* isolates that were non-susceptible to either ertapenem, imipenem, or meropenem, the rate of CPE was 1.4% during 2010–2012^17^, but has increased significantly to 7.6% during 2012–2015^10^. In the present study, the CPE accounted for 29.5% of *E. coli* isolates that were non-susceptible to all of the three carbapenem agents. It should be noted that the difference in the definition of carbapenem non-susceptibility might affect the prevalence of CPE reported in different studies. Nevertheless, increasing carbapenemase-positive *E. coli* isolates is still a call to intensify infection control intervention because the common commensal *E. coli* from the digestive tract might act as a vector to accelerate epidemic dissemination^18^.

*Enterobacteriaceae* carrying *bla*_NDM_ was first identified in Taiwan in 2010 from *K. pneumoniae* in a patient returned from New Delhi where he had been hospitalized for a gunshot injury^8^. Later, the first NDM-1-positive *E. coli* (ST345) was discovered in 2012^9^. NDM has rapidly distributed and become the predominant carbapenemase among *E. coli* during 2012–2015^10^. The present study echoes that NDM are still the predominant carbapenemase in CPEc after year 2015 in Taiwan.

The increase of the NDM-positive *E. coli* isolates in our hospital likely resulted from horizontal gene transfer of the NDM gene carried on mobile genetic elements. Plasmids with similar backbones to the IncX3 *bla*_NDM-5_ and the IncHI2/IncHI2A *bla*_NDM-1_-carrying plasmids found in the present study have been detected in multiple species of the *Enterobacteriaceae*^11,19^. These plasmids are considered important vectors involved in the dissemination of NDM variants in different geographic regions and widespread in different hosts^11,19^. IncX3 plasmids are the most common type of plasmid carrying *bla*_NDM_, mainly in China and its neighboring countries^19^. *E. coli* with *bla*_NDM_-carrying IncX3 plasmids have been found in the livestock animals and poultry in China^7,11,20^. It is of concern that people in community may be exposed to these pathogens through the food chain. NDM-positive *Enterobacteriaceae* in both human and animal guts may serve as the environmental reservoir and source for *bla*_NDM_ dissemination^7,21^.

The presence of mobile genetic element ISAba125 immediate upstream of the NDM-5 gene likely played a role in the mobilization of the NDM-5 gene or its expression of carbapenem resistance^19^. The presence of multiple copies of the NDM-1 gene cassettes in tandem repeat, seen in one of our *bla*_NDM-1_-carrying plasmid, has been reported in a *Klebsiella pneumoniae* isolate KPX^22^. Such phenomenon indicated its active mobilization capability likely due to selective pressure brought about by antibiotic use.

The NDM-positive *E. coli* isolates are mostly genetically diverse with respect to MLST and PFGE in this present study. This is quite different from the spread of KPC-positive *K. pneumoniae* and CTX-M-15-positive *E. coli*, which both disseminates through the successful pandemic clones^23–26^. A total of 15 sequence types (ST) were detected among the 23 CPEc isolates. Among these, ST410 accounted for a majority of NDM-positive *E. coli* (5/9, 55.6%) and all of them showed > 80% similarity by PFGE. Transmission of the ST410 NDM-positive isolates among patients cannot be ruled out since isolates having indistinguishable PFGE patterns were also detected. ST410 *E. coli* has been considered as a reservoir of resistance genes in both human and animals, particularly of the *bla*_CTX-M-15_ gene^27,28^. In China, the carriage of CPEc is also closely associated with specific clones, with ST410 being the second most prevalent among *bla*_NDM_ carrying isolates. Although less common than ST167 and ST617, ST 410 strains with *bla*_NDM_ are widely distributed in several countries and are regarded as a potential emergent high-risk clone for the global dissemination of NDM in *E. coli*^19,29^. Our cgMLST analysis show that the *bla*_NDM_-positive ST410 isolates are highly clonal and similar to those from other countries, which attest to the global dissemination of this carbapenem-resistant *E. coli* lineage^30^.

Colistin remained one of the limited treatment options against CnSE. In our study, the resistant rate to colistin among CnSKP and CnSEc isolates was 16.0% and 5.1%, respectively. CnSE become resistant to colistin via different mechanism, including chromosomal gene mutations and plasmid-borne *mcr-1* genes^31^. The plasmid-mediated *mcr-1* gene raised the concerns that colistin resistance could become more widespread. Tigecycline is another treatment option but decreasing susceptibility rate had been observed in our CPKP (78.6% in 2016 to 58.6% in 2018, data not shown). Prolonged-infusion of high-dose meropenem in combination with other active antimicrobial agent has been proposed for treating CnSE isolates with a meropenem MIC ≤ 8 mg/L^32^. However, in the present study, only 4.6% of CPKP and 8.7% of CPEc isolates display a meropenem MIC ≤ 8 mg/L, which indicates that high-dose prolonged infusion meropenem is not the drug of choice for carbapenemase-producing *E. coli* or *K. pneumoniae*. Several new antimicrobial agents have been developed for carbapenem-non-susceptible bacteria, such as ceftazidime/avibactam, meropenem/vaborbactam and imipenem/relebactam, but none of them have the ability to inhibit metallo-β-lactamases (MBLs), which accounted for 78.2% of CPEc and 28.7% of CPKP in this study. While the prevalence of NDM has increased and become the major carbapenemase in *E. coli*, development of new therapeutic agent is in urgent need because treatment options of MBLs are limited.

There are several limitations to our study. First, this was a single center study with only the two most common CnSE species included. Nevertheless, our study revealed the dissemination of carbapenemase-producing gene among diverse clones in a single center, which should raise the concerns about the emergent resistance from the expansion of multiple bacterial clones and further inter-clonal spread of the resistant determining genes. Second, isolates susceptible to any of the three tested carbapenem would be excluded so we might fail to identify all carbapenemase-positive isolates. Third, harboring carbapenemase-producing genes is not equal to gene expression with carbapenemase production. We regarded them the same because gene expression may be induced under antibiotic pressure even though carbapenemase was not detected.

## Conclusions

In conclusion, the NDM genes appear to disseminate and become the most common carbapenemase among CPEc in Taiwan. The globally distributed *E. coli* strain ST410 accounted for a high proportion of NDM-positive *E. coli* isolates, and the majority of *bla*_NDM_ was found on IncX3 plasmids. Awareness should be raised as the prevalence of NDM-positive *E. coli* might increase rapidly with IncX3 and ST410 being the potential vectors for wide dissemination.

## Methods

### Bacterial isolates and antibiotic susceptibility testing

The study was conducted at National Taiwan University Hospital (NTUH), a major teaching hospital located in northern Taiwan with a total capacity of 2600 beds. Between 2016 and 2018, all clinical *K. pneumoniae* and *E. coli* isolates with non-susceptibility to all of ertapenem, imipenem and meropenem (minimum inhibitory concentrations [MICs] of imipenem and meropenem > 1 μg/ml and MIC of ertapenem > 0.5 μg/ml) were enrolled for subsequent microbiological studies. We used the VITEK 2 Automated System (BioMe ´rieux, Marcy l’Etoile, France) for bacterial identification and for determining the susceptibility to piperacillin/tazobactam, cefmetazole, cefotaxime, ceftazidime, cefepime, ertapenem, meropenem, imipenem, ciprofloxacin, levofloxacin, gentamicin, amikacin, trimethoprime-sulfamethoxazole and tigecycline. Susceptibility to colistin was determined by broth microdilution method as the Clinical & Laboratory Standards Institute (CLSI) suggested^33^. Susceptibilities to colistin and tigecycline were interpreted based on the European Committee on Antimicrobial Susceptibility Testing (EUCAST) guidelines^34^ and the US Food and Drug Administration breakpoint^35^ for *Enterobacteriaceae,* respectively. Susceptibilities to other agents were based on CLSI guidelines^36^. Duplicated isolates were excluded. The study was approved by the NTUH Research Ethics Committee (registration number 201908060RIN) and written or oral informed consent was waived.

### Detection of carbapenemase-encoding genes (CEGs)

DNA was extracted using Viogene DNA extraction kit (Blood Genomic DNA Extraction Midiprep System; Viogene, Taipei, Taiwan) following instruction of the manufacturer. All of the isolates fulfilled the predefined definition of carbapenam nonsusceptibiltiy were subjected to polymerase chain reaction (PCR) detection of genes encoding carbapenemases, including class A (*bla*_KPC_), class B (*bla*_IMP_, *bla*_VIM_, *bla*_NDM_), and class D (*bla*_OXA-48_), using the primers described previously^37,38^. All carbapenemase gene PCR positive amplicons were sequenced to check for amplicon specificity. In this present study, isolates positive for the CEGs were considered as CPE, and those that were negative were considered as non-carbapenemase-producing carbapenem-non-susceptible *Enterobacteriaceae* (non-CP-CnSE).

### Molecular typing

To analyze the clonal relatedness, the CPE isolates were subject to pulsed-field gel electrophoresis (PFGE) and multi-locus sequence typing (MLST) following previously published protocols and information from the MLST website (http://bigsdb.pasteur.fr/ecoli/ecoli.html and https://bigsdb.pasteur.fr/klebsiella/primers_used.html)^39^. Briefly, PFGE was carried out with genomic DNA prepared in agarose plugs and digested with the restriction enzyme Spel (New England Biolabs, NEB, Beverly, Mass., U.S.A.). The Spel-digested DNA was separated by PFGE in a 1% agarose gel (SeaKem Gold Agarose, Lonza Rockland, Inc., ME, U.S.A.) in 0.5 × TBE buffer (45 mM Tris, 45 mM boric acid, and 1.0 mM EDTA, pH 8.0) for 22 h at 6 V/cm at a temperature of 14 °C, with a linearly ramped pulse times of 1.79–18.66 s using a CHEF Mapper apparatus (Bio-Rad Laboratories, Richmond, CA). For interpretation of the PFGE banding patterns, unweighted-pair group method with arithmetic mean (UPGMA) dendrograms were constructed from the original data (Bionumerics version 6.6, Applied Maths, Sint-Martens-Latem, Belgium). Isolates that exhibited similarity of ≥ 80% of their banding patterns were considered as closely related strains.

### Whole genome sequencing of NDM-positive *E. coli* isolates

Whole genome sequencing (WGS) was performed on all NDM-positive *E. coli* isolates (5 NDM-5 and 4 NDM-1-positive). De novo sequencing of each of the *E. coli* strain was conducted by a shotgun approach using Oxford Nanopore MinION rapid 1D sequencing and illumina 2 × 150-bp pair-end shotgun reads. The long reads and short reads were assembled using Unicycler following the program operating instructions^40^. Annotation of the genomes was performed using RAST^41^. Subsequent analyses of the assembled genomes were performed using CLC genomic workbench (Qiagen, Düsseldorf, Germany) and MAUVE^42^.

### Consumption of carbapenems

The data of carbapenem consumption at NTUH were obtained for the years 2010–2018 in order to observe the annual fluctuation. The consumption of each carbapenem was provided in the form of defined daily dose (DDD) per 1000 patient-days. DDD is the assumed average treatment dose per day for a drug prescribed for its main indication in adults, as defined by the World Health Organization (WHO)^43^.

### Statistical analysis

Statistical analysis was performed using STATA software (version 11, StataCorp, College Station, TX). The χ2 test was used for categorical variables. Carbapenem consumption and the prevalence of CPE during every half year period were assessed for trends by utilizing the chi-square test for trend. Correlations between CPE incidence and carbapenem consumption were assessed with Spearman’s correlations, using total carbapenem consumption one year prior to the predefined time period. A value of *p* < 0.05 was considered to be statistically significant.

### Ethics approval and consent to participate

The study was approved by the NTUH Research Ethics Committee (registration number 201908060RIN) and written or oral informed consent was waived.

## Supplementary Information

Supplementary Information.

## Data Availability

The datasets used and/or analyzed during the current study are available from the corresponding author on reasonable request.
