# Supplementary material for: Increasing New Delhi metallo-β-lactamase-positive Escherichia coli among carbapenem non-susceptible Enterobacteriaceae in Taiwan during 2016 to 2018
Source: Sci Rep. 2021 Jan 28;11:2609. doi: 10.1038/s41598-021-82166-8 (PMC7843600; doi:10.1038/s41598-021-82166-8)

**SUPPLEMENTAL FILES:**

**Increasing New Delhi Metallo-β-lactamase-Positive *Escherichia coli* Among Carbapenem Non-susceptible *Enterobacteriaceae* in Taiwan During 2016 to 2018**

Yu-Shan Huang,^1,2^ Wan-Chen Tsai,^3^ Jia-Jie Li,^4^ Pao-Yu Chen,^1,2^ Jann-Tay Wang,^1*^ Ying-Tsong Chen,^5^ Feng-Jui Chen,^6^ Tsai-Ling Lauderdale,^6*^ Shan-Chwen Chang^1^

^1^Department of Internal Medicine, National Taiwan University Hospital, Taipei 100, Taiwan

^2^Graduate Institute of Clinical Medicine, National Taiwan University College of Medicine, Taipei, Taiwan

^3^Department of Internal Medicine, National Taiwan University Hospital Biomedical Park Hospital, Department of Internal Medicine

^4^Taipei Municipal Jianguo senior high school, Taipei, Taiwan

^5^Institute of Molecular and Genomic Medicine, National Health Research Institutes, Miaoli County, Taiwan

^6^National Institute of Infectious Diseases and Vaccinology, National Health Research Institutes, Zhunan, Taiwan

**Corresponding author:**

Jann-Tay Wang, M.D., Ph.D.

Department of Internal Medicine, National Taiwan University Hospital

7 Chung-Shan South Road, Taipei, Taiwan 100

E-mail address: wang.jt1968@gmail.com

Telephone: +886-2-23123456 Ext. 63517

**Supplementary Tables**

**Supplementary Table 1.** Distribution of minimum inhibitory concentrations (MICs) among carbapenem-non-susceptible *E. coli* and *K. pneumoniae* isolates

| Antimicrobial agents | Carbapenem-non-susceptible  *E. coli* (n=78) | | | Carbapenem-non-susceptible  *K. pneumoniae* (n=306) | | |
| --- | --- | --- | --- | --- | --- | --- |
|  | MIC_50_ | MIC_90_ | Range | MIC_50_ | MIC_90_ | Range |
| Cefotaxime | ≥64 | ≥64 | ≤1 - ≥64 | ≥64 | ≥64 | ≤1 - ≥64 |
| Ceftazidime | ≥64 | ≥64 | 4 - ≥64 | ≥64 | ≥64 | ≤1 - ≥64 |
| Cefmetazole | ≥64 | ≥64 | ≤1 - ≥64 | ≥64 | ≥64 | ≤1 - ≥64 |
| Cefepime | 16 | ≥64 | ≤1 - ≥64 | ≥64 | ≥64 | ≤1 - ≥64 |
| Amikacin | ≤2 | 16 | ≤2 - ≥64 | ≤2 | ≥64 | ≤2 - ≥64 |
| Gentamicin | 4 | ≥16 | ≤1 - ≥16 | ≥16 | ≥16 | ≤1 - ≥16 |
| Ciprofloxacin | ≥4 | ≥4 | ≤0.25-≥4 | ≥4 | ≥4 | ≤0.25-≥4 |
| Levofloxacin | ≥8 | ≥8 | ≤0.12 - ≥8 | ≥8 | ≥8 | ≤0.12 - ≥8 |
| Tigecycline | ≤0.5 | 1 | ≤0.5 - ≥8 | 2 | ≥8 | ≤0.5 - ≥8 |
| Pip/Tazo | ≥128 | ≥128 | 16 - ≥128 | ≥128 | ≥128 | 4 - ≥128 |
| TMP/SMX | ≥320 | ≥320 | ≤20 - ≥320 | ≥320 | ≥320 | ≤20 - ≥320 |
| Colistin | 1 | 2 | 0.5 - 4 | 1 | 8 | ≤0.25-≥125 |

**Abbreviations**: MICs, minimal inhibitory concentrations; Pip/Tazo, piperacillin/tazobactam; TMP/SMX, trimethoprim/sulfamethoxazole

# **Supplementary Table 2.** Detailed susceptibilities of carbapenemase-producing *E. coli* and *K. pneumoniae* by various types of carbapenemase

| Antimicrobial agents | Carbapenemase-producing *E. coli*  (%Susceptible) | | | | Carbapenemase-producing *K. pneumoniae* (%Susceptible) | | | |
| --- | --- | --- | --- | --- | --- | --- | --- | --- |
|  | NDM  (n=9) | IMP-8  (n=7) | KPC  (n=5) | VIM-1  (n=2) | NDM  (n=3) | IMP-8  (n=5) | KPC  (n=78) | VIM-1  (n=24) |
| Cefotaxime | 0 | 0 | 0 | 0 | 0 | 0 | 0 | 0 |
| Ceftazidime | 0 | 0 | 0 | 0 | 0 | 0 | 0 | 0 |
| Cefmetazole | 0 | 0 | 40.0 | 0 | 0 | 0 | 1.3 | 12.5 |
| Cefepime | 0 | 0 | 40.0 | 0 | 0 | 0 | 1.3 | 8.3 |
| Amikacin | 88.9 | 100 | 100 | 100 | 100 | 100 | 91.0 | 87.5 |
| Gentamicin | 33.3 | 71.4 | 40.0 | 100 | 66.7 | 100 | 25.6 | 50.0 |
| Ciprofloxacin | 11.1 | 14.3 | 20.0 | 50.0 | 33.3 | 40.0 | 0 | 45.8 |
| Levofloxacin | 22.2 | 14.3 | 20.0 | 50.0 | 33.3 | 40.0 | 0 | 45.8 |
| Tigecycline | 100 | 100 | 100 | 100 | 66.7 | 40.0 | 67.5 | 50.0 |
| Pip/Tazo | 0 | 14.3 | 0 | 0 | 0 | 0 | 0 | 0 |
| TMP/SMX | 22.2 | 28.6 | 20.0 | 0 | 33.3 | 40.0 | 38.5 | 16.7 |
| Colistin | 100 | 100 | 100 | 100 | 100 | 100 | 78.2 | 79.2 |

**Abbreviations**: Pip/Tazo, piperacillin/tazobactam; TMP/SMX, trimethoprim/sulfamethoxazole

**Supplementary Figures**

# **Supplementary Figure 1.** Comparison of the plasmids from NDM-1-positive eco286, NDM-1-positive eco314, and MG878867


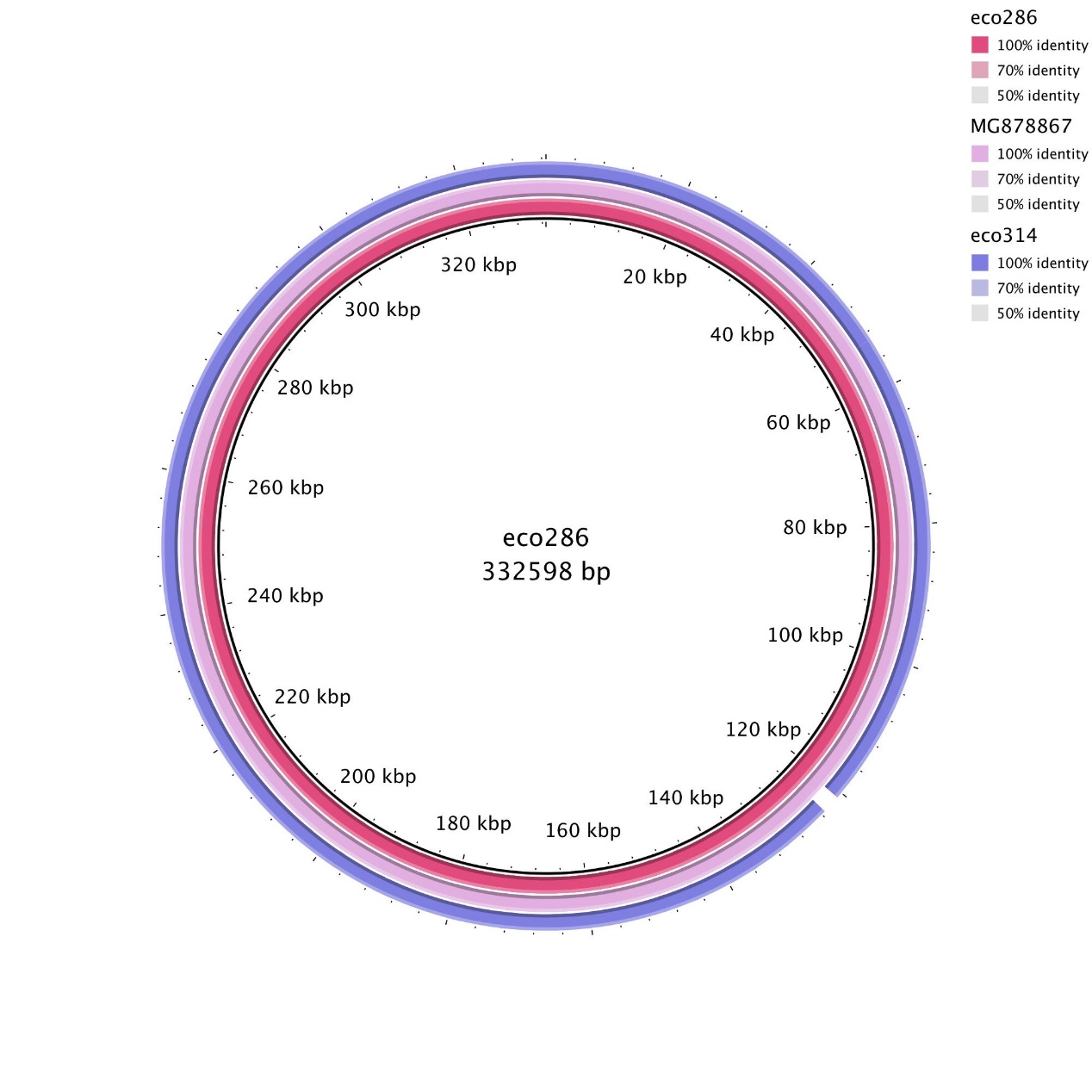


# **Supplementary Figure 2.** Annual carbapenem consumption from 2010 to 2018 in National Taiwan University Hospital


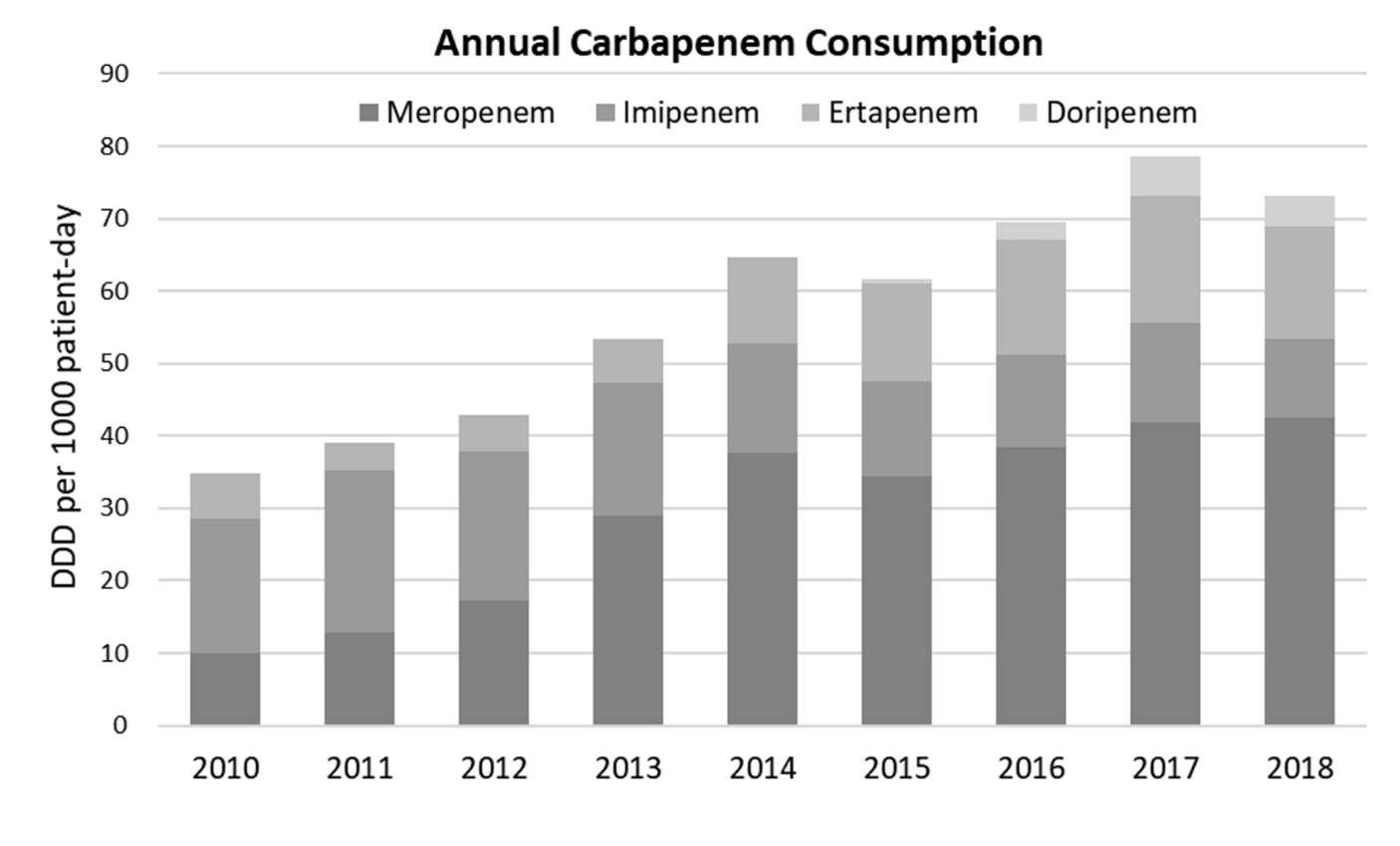

Supplement: Supplementary file 1 — Supplementary Information. [file 41598_2021_82166_MOESM1_ESM.docx]
